# A new justification of the Hartung‐Knapp method for random‐effects meta‐analysis based on weighted least squares regression

**DOI:** 10.1002/jrsm.1356

**Published:** 2019-08-14

**Authors:** Robbie C. M. van Aert, Dan Jackson

**Affiliations:** ^1^ Methodology and Statistics Tilburg University Tilburg Netherlands; ^2^ Statistical Innovation Group, Advanced Analytics Centre AstraZeneca Cambridge United Kingdom

**Keywords:** Hartung‐Knapp modification, meta‐analysis, meta‐regression, random‐effects weighted least squares regression

## Abstract

The Hartung‐Knapp method for random‐effects meta‐analysis, that was also independently proposed by Sidik and Jonkman, is becoming advocated for general use. This method has previously been justified by taking all estimated variances as known and using a different pivotal quantity to the more conventional one when making inferences about the average effect. We provide a new conceptual framework for, and justification of, the Hartung‐Knapp method. Specifically, we show that inferences from fitted random‐effects models, using both the conventional and the Hartung‐Knapp method, are equivalent to those from closely related intercept only weighted least squares regression models. This observation provides a new link between Hartung and Knapp's methodology for meta‐analysis and standard linear models, where it can be seen that the Hartung‐Knapp method can be justified by a linear model that makes a slightly weaker assumption than taking all variances as known. This provides intuition for why the Hartung‐Knapp method has been found to perform better than the conventional one in simulation studies. Furthermore, our new findings give more credence to ad hoc adjustments of confidence intervals from the Hartung‐Knapp method that ensure these are at least as wide as more conventional confidence intervals. The conceptual basis for the Hartung‐Knapp method that we present here should replace the established one because it more clearly illustrates the potential benefit of using it.

## 1 INTRODUCTION

The  random‐effects model for meta‐analysis is commonly used to synthesize independent effect size estimates with underlying heterogeneous true effect sizes.^1^, ^2^ Two parameters are estimated in the random‐effects model: the average effect and the between‐study variance (the variance of the studies' true effect sizes). When using standard methods for meta‐analysis, the between‐study variance is first estimated. This estimate is then, together with the studies' within‐study sampling variances, incorporated into the study weights when estimating the average effect and making inferences regarding this parameter. This standard approach for performing meta‐analyses provides a pooled estimate that is a weighted average of the estimated effects. However, estimates of the between‐study variance are often imprecise, especially if the number of studies in a meta‐analysis is small.^3^, ^4^, ^5^ This uncertainty is ignored when making inferences under the random‐effects model using standard methodologies  and so relying on a normal approximation for the average effect, with the potential risk of making inaccurate statistical inferences. A further and related concern is that the within‐study variances are often imprecisely estimated if the studies are small (and these variances may also be correlated with the corresponding study specific estimated effects). This uncertainty (and correlation) is also conventionally ignored when fitting the random‐effects model, which can have detrimental consequences when making statistical inferences.^6^, ^7^, ^8^, ^9^, ^10^

Hartung and Knapp^11^, ^12^, ^13^ and Sidik and Jonkman^14^ propose a “modified” or “refined” method (henceforth called the Hartung‐Knapp method) for making inferences about the average effect when fitting the random‐effects model. The Hartung‐Knapp method uses quantiles of the *t* distribution rather than the standard normal distribution in the more conventional method when computing a confidence interval (CI) for the average effect. This is justified by multiplying the conventional variance of the estimated average effect with a scaling factor, because (treating the within and between‐study variances as known) the proposed pivot for making inferences then follows a *t* distribution.^11^ However, we will see later that the Hartung‐Knapp method can in fact be justified by a slightly weaker assumption than this, indeed this is the main point made by our new justification of this method.

Simulation studies suggest that the Hartung‐Knapp method is a substantial improvement over the more conventional method, in the sense that the actual coverage probability of 95% CIs for the average effect has been found to be closer to the nominal level.^15^, ^16^, ^17^ The calculations required by these two methods are closely related, and it is difficult to obtain evidence concerning which method is best without resorting to simulation studies. To our knowledge, the only analytical evidence supporting claim that the Hartung‐Knapp method performs better is provided by Sidik and Jonkman^18^ who show, under the assumptions of the random‐effects model, that the coverage probability of the CI from the Hartung‐Knapp method is exact in the artificial setting where all within‐study variances are the same and the random‐effects model is true. Simulation studies provide evidence, but little or no intuition, for the greater accuracy of the inference from the Hartung‐Knapp method in more realistic settings. These findings indicate that the Hartung‐Knapp method possesses different properties to the conventional method, and so these two methods are perhaps most naturally conceptualised as being completely alternative statistical methods that are both valid under the fitted random‐effects model.^19^

Given its greater accuracy, it is therefore perhaps surprising that the Hartung‐Knapp method has not been more widely adopted. However, an undesirable feature of this method is that its CI for the average effect can be shorter than the corresponding conventional CI for some datasets.^16^, ^19^ This is unsatisfactory because the more conventional method ignores the uncertainty in the unknown variance components, and so its CI can be anticipated to be too short in many settings. The suggestion to accompany results from the Hartung‐Knapp method with conventional fixed‐effect^16^ or random‐effects^19^ meta‐analyses as a sensitivity analysis is one response to the concern that the CI based on the Hartung‐Knapp method can be too short. Another response to this concern is to propose ad hoc adjustments of the Hartung‐Knapp method that ensure that the width of its CI are at least as wide as the conventional CI.^19^, ^20^ As we will see below, this type of ad hoc adjustment is easily made by constraining a scaling factor to be greater than or equal to one. A consequence of our new findings will be to give further credence to methods that constrain this scaling factor in this and other ways.

The main aim of this paper is to provide intuition for why the Hartung‐Knapp method has been found to be more accurate in simulation studies by establishing a new conceptual framework for it. This framework shows that the Hartung‐Knapp method can be justified using standard regression based methods that allow us to make a slightly weaker assumption than treating all variance components as if known; more specifically, we will see that the Hartung‐Knapp method can be justified by an intercept only linear regression where the total study variances are assumed to be known only to within a constant of proportionality. However, the conventional method does not allow us to make this slightly weaker assumption either when justified in the usual way or by using our new framework. Comparing the conventional with the Hartung‐Knapp method in this way explains why the latter method has been found to be more accurate in simulation studies. This and the other insights that follow are not at all obvious from the established way to justify the Hartung‐Knapp method. We therefore suggest that our new framework for justifying this method should replace the one that is currently used.

The rest of the paper is set out as follows. We continue in section two by introducing the random‐effects model and describing the conventional and Hartung‐Knapp methods for making inferences for the average effect. We also give the current standard derivations that justify the use of these two methods. In Section  3, we describe weighted least squares (WLS) regression in general and two WLS regression models in particular that differ in the assumption that is made regarding the error variances. We establish the equivalences between the conventional and Hartung‐Knapp methods for random‐effects meta‐analysis and these WLS regression models in Section  4, where we also extend our results to include random‐effects meta‐regression models. In Section  5, we illustrate our findings numerically using two real examples. We describe further insights afforded by our new conceptual framework in Section  6, and the paper ends with a short discussion in Section  7.

## 2 THE CONVENTIONAL AND HARTUNG‐KNAPP METHODS FOR MAKING INFERENCES FOR THE AVERAGE EFFECT UNDER THE RANDOM‐EFFECTS MODEL

We will start by describing the conventional random‐effects model for meta‐analysis following Chapter 12 of Borenstein et al.^2^ This model justifies the use of the conventional and Hartung‐Knapp methods to make inferences about the average effect. The same average effect is estimated when using both of these methods. Hence, the Hartung‐Knapp method does not modify the point estimate in any way and so does not address any concerns related to bias in this parameter. However, different results are obtained when making further inferences (such as performing hypothesis tests and calculating confidence intervals) about the average effect. Both the conventional and Hartung‐Knapp methods could be supplemented with the same CI for the between‐study variance.^21^, ^22^

The random‐effects model can be written as (for example, see Equation 12.1 in Borenstein et al 2 where we slightly adapt the notation to avoid a notational conflict later)

(1) $$Y_{i}=\mu +\varsigma_{i}+\delta_{i},\;$$

where *Y*_*i*_, *i*=1, …, *n*, is the estimated effect size from the *i*th study, *μ* is the average effect, *ς*_*i*_ is a random effect denoting the difference between *μ*, the *i*th study's true effect size, and *δ*_*i*_ is the within‐study sampling error. It is usually assumed that *ς*_*i*_∼*N*(0,*τ* ^2^), where *τ* ^2^ is the between‐study variance in true effect sizes, but some estimation methods avoid assuming the *ς*_*i*_ and *δ*_*i*_ are normally distributed.^23^, ^24^ We further assume that $\delta_{i}∼N(0,s_{i}^{2})$, where $s_{i}^{2}$ is the within‐study sampling variance of the *i*th study. The sampling variances $s_{i}^{2}$ are usually estimated in practice and then regarded as known under the random‐effects model. Moreover, all *ς*_*i*_ and *δ*_*i*_ are assumed to be mutually independent. Fitting the random‐effects model therefore requires the estimation of two remaining parameters, *μ* and *τ* ^2^.

Many estimators have been developed for estimating *τ* ^2^ (see Veroniki et al 25 and Langan et al 26 for an overview). Any of these estimators of *τ* ^2^ may be used when computing the weights, $w_{i}^{*}=1/\left(s_{i}^{2}+{\hat{\tau }}^{2}\right)$ and in conjunction with either the conventional and Hartung‐Knapp methods when making inferences about *μ*. These inferences are approximate rather than exact, because in conventional justifications all variances $s_{i}^{2}$ and ${\hat{\tau }}^{2}$ are treated as fixed and known when making inferences for the average effect under random‐effects model.^1^ This is the case for both the conventional and Hartung‐Knapp methods that follow, and may have especially detrimental consequences for meta‐analyses containing a small number of studies when making statistical inferences.^27^, ^28^ The average effect *μ* is estimated using traditional inverse variance weights^2^ of $w_{i}^{*}$ when using both the conventional and Hartung‐Knapp methods. In either case, the average effect is therefore estimated as (see Equation 12.7 in Borenstein et al^2^ with some changes in notation here)

(2) $$\hat{\mu }=\frac{\sum w_{i}^{*}Y_{i}}{\sum w_{i}^{*}}.$$

### 2.1 The conventional method for making inference for *μ* under the random‐effects model

The variance of $\hat{\mu }$, under the fitted random‐effects model, where we treat all variances as fixed and known, is traditionally computed as (see Equation 12.8 in Borenstein et al^2^)

(3) $$V_{\hat{\mu }}=\frac{1}{\sum w_{i}^{*}}.$$

The standard error of $\hat{\mu }$ for the conventional method is therefore $\sqrt{V_{\hat{\mu }}}$. The null hypothesis, *H*_0_:*μ*=0, is tested using the test statistic, $\hat{\mu }/\sqrt{V_{\hat{\mu }}}$, by comparing this to critical values of the standard normal distribution (see Equation 12.12 in Borenstein et al^2^). A CI for *μ* is calculated as $\hat{\mu }\pm z_{\alpha /2}\sqrt{V_{\hat{\mu }}}$, where *z*_*α*/2_ is the (1−*α*/2) quantile of the standard normal distribution, with *α*=0.05, and so *z*_*α*/2_≈1.96, for a 95% CI (see Equations 12.10 and 12.11 in Borenstein et al^2^). These conventional inferences are obtained from the approximate pivot

(4) $$\frac{\hat{\mu }-\mu }{\sqrt{V_{\hat{\mu }}}}∼N(0,1),\;$$

where *N*(0,1) denotes the standard normal distribution.

### 2.2 The Hartung‐Knapp method for making inference for *μ* under the random‐effects model

Hartung and Knapp^11^, ^12^, ^13^ and Sidik and Jonkman^14^ propose using an alternative pivot for making inferences about *μ*. Our exposition of this method follows Hartung and Knapp,^13^ but we adapt their notation to make it equivalent with the notation used above.

An alternative estimator for the variance of $\hat{\mu }$ (see Equation 10 of Hartung and Knapp^13^) is

$$V_{\hat{\mu }}^{HK}=\frac{1}{n-1}\sum \frac{w_{i}^{*}}{w_{+}^{*}}{\left(Y_{i}-\hat{\mu }\right)}^{2},$$

where $w_{+}^{*}=\sum w_{j}^{*}$. This estimator of the variance of $\hat{\mu }$ is the conventional estimator $V_{\hat{\mu }}$ scaled by *H* ^*2^, where

(5) $$V_{\hat{\mu }}^{HK}=\left(\frac{\sum w_{i}^{*}{\left(Y_{i}-\hat{\mu }\right)}^{2}}{n-1}\right)V_{\hat{\mu }}=H^{*2}V_{\hat{\mu }}.$$

The standard error of $\hat{\mu }$ for the Hartung‐Knapp method is therefore $\sqrt{V_{\hat{\mu }}^{HK}}$. If ${\hat{\tau }}^{2}=0$, then *H* ^*2^ is equal to the *H* ^2^ statistic proposed by Higgins and Thompson^29^ for quantifying the between‐study heterogeneity. The null hypothesis, *H*_0_:*μ*=0, is tested using the test statistic (see Equation 11 in Hartung and Knapp^13^), $\hat{\mu }/\sqrt{V_{\hat{\mu }}^{HK}}$, by comparing this to critical values of the *t* distribution with *n*−1 degrees of freedom.^11^ A CI for *μ* is calculated as $\hat{\mu }\pm t_{n-1,\alpha /2}\sqrt{V_{\hat{\mu }}^{HK}}$, where *t*_*n*−1,*α*/2_ is the (1−*α*/2) quantile of the *t* distribution with *n*−1 degrees of freedom. These inferences using the Hartung‐Knapp method are obtained from the approximate pivot

(6) $$\frac{\hat{\mu }-\mu }{\sqrt{V_{\hat{\mu }}^{HK}}}∼t_{n-1},\;$$

where *t*_*n*−1_ denotes the *t* distribution with *n*−1 degrees of freedom. Comparing Equations   (4) and (6), we can see that the conventional and Hartung‐Knapp methods use different pivots when making inferences for *μ* under the random‐effects model.

### 2.3 The usual justifications of the conventional and Hartung‐Knapp methods

The usual formal justifications of the conventional and Hartung‐Knapp methods treat all variances as if they are known. That is, we assume that $\tau^{2}={\hat{\tau }}^{2}$ and $s_{i}^{2}$ is the true within‐study variance for all *i* (or, at least, that replacing these parameters by their estimates is a reasonable approximation; see Jackson^30^ for formal justifications of these approximations). In practice, all variance components are estimated before making inferences for *μ*, which means that both of these methods are merely approximate. Treating all variances as known also means that we can treat the $w_{i}^{*}$ as known.

The distributional results in Equations   (4) and (6) that motivate the conventional and Hartung‐Knapp methods then follow from standard statistical theory. Briefly, for the Hartung‐Knapp method this is because the approximate pivot in Equation   (6) can be written as $P=Z/\sqrt{H^{*2}}$, where (by taking all variances to be known) $Z=(\hat{\mu }-\mu )/\sqrt{V_{\hat{\mu }}}∼N(0,1)$ and $(n-1)H^{*2}∼\chi_{n-1}^{2}$, where *Z* and *H* ^*2^ are independent. Hence,  *P*∼*t*_*n*−1_. The approximate pivot in Equation   (4) used in the conventional method is directly justified by the distribution of *Z* in this argument.

## 3 WLS REGRESSION

As we have seen, the conventional and Hartung‐Knapp methods for random‐effects meta‐analysis are justified by the approximate pivots in Equations   (4) and (6), respectively. However, we will see below that these two methods can also be justified using the standard statistical theory of WLS regression. Upon conceptualising these two methods for meta‐analysis as applications of WLS regression further insight will be possible.

In order to establish this new link between WLS regression models and the two methods for meta‐analysis described above, we begin by describing the theory of WLS regression. We primarily use Section 4.1.2 of the book by Fahrmeir et al^31^ to describe WLS regression, but we will also refer to a variety of other standard textbooks.^32^, ^33^, ^34^, ^35^, ^36^ The notation of Fahrmeir et al^31^ is slightly adapted here to avoid a clash of notation with earlier sections of this paper.

In a WLS regression model, we assume that the response variable *Y*_*i*_, *i*=1,2,…*n*, depends on one or more predictor variables that are fully observed. The WLS regression model is

(7) Y=X𝛃+ϵ,

where ***Y*** is a *n*×1 column vector containing *Y*_*i*_, *i*=1, …, *n*, ***X*** is the *n*×*p* design matrix with *p* denoting the number of regression parameters (sometimes also referred to as the model matrix), ***β*** is a *p*×1 column vector of *p* regression parameters, and ***ϵ*** is a *n*×1 column vector containing the sampling errors. We assume that E(ϵ)=0, where ***ϵ*** is taken to follow a multivariate normal distribution. Different assumptions about the form of the covariance matrix Var(***ϵ***) result in different types of WLS regression models. The WLS regression model reduces to the ordinary least squares regression model if all variances (ie, elements on the diagonal of the covariance matrix Var(***ϵ***)) are equal to each other and the observations are uncorrelated (ie, off diagonal elements of the covariance matrix Var(***ϵ***) equal to zero).^35^ Equal variances is also referred to as homoscedasticity that is in contrast with heteroscedasticity where the variances are not the same. We fully examine two particular forms of the covariance matrix Var(***ϵ***) in detail immediately below, and we will subsequently show that these can be used to motivate the conventional and Hartung‐Knapp methods for random‐effects meta‐analysis.

### 3.1 Known variances and independent errors

If the error variances are assumed to be known, and the errors are assumed to be independent, then we have $\epsilon_{i}∼N(0,\sigma_{i}^{2})$, where these *ϵ*_*i*_ are the entries of ***ϵ*** in model (7) and $\sigma_{i}^{2}$ are the error variances. In this model, all $\sigma_{i}^{2}$ are known, and so are fixed constants, and the *ϵ*_*i*_ are independent. WLS regression models with known variances are rarely applied in practice, because it is usually unrealistic to assume that variances are known without error. Perhaps for this reason, this type of model is not discussed in Fahrmeir et al^31^ but is described in other books on linear models.^33^

Let ***W***=diag $\left(1/\sigma_{i}^{2}\right)$ denote the *n*×*n* diagonal matrix containing the weights $1/\sigma_{i}^{2}$, so that under the model, we have Var(***ϵ***)=***W*** ^−1^. The variances $\sigma_{i}^{2}$ are assumed to be known so that ***W*** is also treated as known. These weights are optimal under the model, because they result in the best linear unbiased estimation of the regression coefficients.^36^ The regression parameters ***β*** are estimated as (see Equation 11.9 in Kutner et al^33^

(8) $$\hat{𝛃}={\left(X^{T}WX\right)}^{-1}X^{T}Wy,\;$$

where ***y*** is the observed value of ***Y***. Searle (1971) gives a more general result than Equation   (8) on his page 87, where Equation   (8) is given as the estimate where ***W*** ^−1^=Var(***ϵ***), for example, in Searle's result, the *ϵ*_*i*_ need not be independent so that ***W*** is not a diagonal matrix. Then, from model (7) and estimating Equation   (8), together with Var(***ϵ***)=***W*** ^−1^ and the standard result that Var(***MX***)=***M***Var(***X***)***M*** ^*T*^, where ***M*** is a matrix of constants, we have the standard result

(9) $$\mathrm{Var}(\hat{𝛃})={\left(X^{T}WX\right)}^{-1}.$$

Inference using Equations   (8) and (9) is then straightforward. This is because matrices ***X*** and ***W*** contain fixed constants so that, from Equation   (8), $\hat{𝛃}$ is a linear combination of the multivariately normally distributed (with known variance) ***y***, so that $\hat{𝛃}$ is also multivariate normal with known variance. The null hypothesis *H*_0_:*β*_*j*_=0 is therefore tested using the test statistic ${\hat{\beta }}_{j}/\sqrt{\mathrm{Var}({\hat{\beta }}_{j})}$, where ${\hat{\beta }}_{j}$ is the *j*th entry of $\hat{𝛃}$ from Equation   (8) and $\mathrm{Var}({\hat{\beta }}_{j})$ is the entry in the *j*th row and column of $\mathrm{Var}(\hat{𝛃})$ from Equation   (9). We compare this test statistic to critical values of the standard normal distribution when performing hypothesis testing. A CI for ${\hat{\beta }}_{j}$ is calculated as ${\hat{\beta }}_{j}\pm z_{\alpha /2}\sqrt{Var({\hat{\beta }}_{j})}$ where *z*_*α*/2_ is the (1−*α*/2) quantile of the standard normal distribution.^33^

### 3.2 Error variances known up to constant of proportionality

Popular software packages that can be used for fitting WLS regression models (ie, SPSS,^37^ SAS,^38^ and R^39^) do not by default fit a model where the error variances are assumed to be known as in the previous section. These software packages usually assume that the error variances are known up to a constant of proportionality that is a weaker and more realistic assumption than assuming that the error variances are known. We now assume a much more standard model of this type.

In our second model, we assume that ***ϵ***∼*N*(**0**,*k* ***W*** ^−1^), where *k* is the unknown constant of proportionality and ***W*** continues to be the diagonal matrix containing the weights $1/\sigma_{i}^{2}$. These weights, and so ***W***, are treated as known. Hence, we assume $\epsilon_{i}∼N\left(0,k\sigma_{i}^{2}\right)$ where all *ϵ*_*i*_ are independent. If we further assume that *k*=1, we obtain the model with known variances and independent errors as described above, but in this section,  *k* is another unknown that must be estimated. Hence, this second WLS regression model is a slightly more general model than the first and is more commonly used in practice.

An important observation is that the regression parameters continue to be estimated using Equation   (8). This follows from the more general result of Searle^35^that, as explained above, states that Equation   (8) applies more generally provided that ***W*** ^−1^=Var(***ϵ***). Then, any constant of proportionality *k* that is applied to Var(***ϵ***), so that the constant *c*=1/*k* is applied to its inverse ***W***, immediately cancels from Equation   (8).^33^ This means that $\hat{𝛃}$ is the same regardless of whether or not the variances are treated as known or instead known up to a proportionality constant; conceptually, the constant of proportionality does not change the relative weight that each observation receives in the WLS regression model.

However, from model (7) and estimating Equation   (8), together with ***ϵ***∼*N*(**0**,*k* ***W*** ^−1^), we now have^31^, ^32^, ^33^

(10) $$\mathrm{Var}(\hat{𝛃})=k{\left(X^{T}WX\right)}^{-1}.$$

Comparing Equations   (9) and (10), we can see that the value of *k* affects the precision of the estimation in the way we should expect; as *k* increases so does the residual variance of ***ϵ*** and so Var $\left(\hat{𝛃}\right)$ also increases in *k*.

The proportionality constant *k* is unknown, but is conventionally estimated as the weighted mean squared error (MSE)

(11) $$\hat{k}=\frac{1}{n-p}{\hat{\epsilon }}^{T}W\;\hat{\epsilon ,}\;$$

where $\hat{\epsilon }$ is the column vector containing the observed residuals.^31^, ^32^, ^33^ We then substitute the estimate of *k* from Equation   (11) into Equation   (10) in order to estimate $\mathrm{Var}(\hat{𝛃})$. A more general result than we require for making inferences that considers an arbitrary linear combination of regression parameters is given in Section 7.2.2 of Yan and Su.^32^ The standard methods for making inferences that follow take this linear combination to be *β*_*j*_. As in the previous model, the null hypothesis *H*_0_:*β*_*j*_=0 is tested using the test statistic, ${\hat{\beta }}_{j}/\sqrt{\mathrm{Var}({\hat{\beta }}_{j})}$, where $\mathrm{Var}({\hat{\beta }}_{j})$ is the entry in the *j*th row and column of the estimated $\mathrm{Var}(\hat{𝛃})$ from Equation   (10) with *k* replaced by its estimate in Equation   (11). However, we now compare this test statistic to critical values of the *t* distribution with *n*−*p* degrees of freedom when performing hypothesis testing. A CI for ${\hat{\beta }}_{j}$ is calculated as ${\hat{\beta }}_{j}\pm t_{n-p,\alpha /2}\sqrt{\mathrm{Var}({\hat{\beta }}_{j})}$ where *t*_*n*−*p*,*α*/2_ is the (1−*α*/2) quantile of the *t* distribution with *n*−*p* degrees of freedom.^32^, ^35^

## 4 EQUIVALENCES BETWEEN RANDOM‐EFFECTS MODEL FOR META‐ANALYSIS AND WLS REGRESSION MODELS

We have now described two alternative methods for making inferences under the random‐effects model for meta‐analysis (the conventional and Hartung‐Knapp methods) and two alternative WLS regression models (error variances assumed known and error variances assumed known up to a constant of proportionality). We anticipate that some parallels between the meta‐analysis and WLS regression methodologies that we have presented may already be apparent to the reader, so that the connections we will ultimately make may be unsurprising. For example, the conventional and Hartung‐Knapp methods for random‐effects meta‐analysis result in the same estimate $\hat{\mu }$ and both types of the WLS regression model result in the same $\hat{𝛃}$. Furthermore, the standard normal distribution is used when making inferences using the conventional method for meta‐analysis and our first WLS regression model (error variances known), whereas the *t* distribution is used when using the Hartung‐Knapp method for meta‐analysis and our second WLS regression model (error variances known up to a constant of proportionality). Finally, the scaling factor *H* ^*2^ in the Hartung‐Knapp method for meta‐analysis is a weighted MSE term where the weighted sum of squares is divided by its associated degrees of freedom, and the proportionality constant *k* in the second WLS regression model is also estimated in this way. Moreover, these weighted MSEs are multiplied by variances from the conventional meta‐analysis and the first of our WLS regression models, in order to provide these variances when using the corresponding alternative method and model. We will now formally establish links between standard methods for meta‐analysis and WLS regression models.

### 4.1 WLS regression and the conventional method for meta‐analysis

Let us consider the most simple form of the WLS regression model in Equation   (7) where there are no predictors (and so we have an intercept only model). Let us also assume known variances and independent errors as in Section  3.1, but we now assume that $\epsilon_{i}∼N(0,s_{i}^{2}+{\hat{\tau }}^{2})$. Hence, in the notation of Section  3.1, we have $\sigma_{i}^{2}=s_{i}^{2}+{\hat{\tau }}^{2}$. When using the conventional method for meta‐analysis, the $s_{i}^{2}$ and ${\hat{\tau }}^{2}$ are estimated but treated as known when making inferences about the average effect. Hence, treating the $\sigma_{i}^{2}=s_{i}^{2}+{\hat{\tau }}^{2}$ as known in our regression model mimics the conventional approximations used in meta‐analysis.

Hence,  ***X***=**1**, where **1** is an *n*×1 column vector where every entry is 1 and $W=\text{diag}\left(1/\left(s_{i}^{2}+{\hat{\tau }}^{2}\right)\right)$. Equations  (8) and (9) then provide

$$\hat{\beta }={\left(1^{T}W1\right)}^{-1}1^{T}Wy,$$

and

$$Var(\hat{\beta })={\left(1^{T}W1\right)}^{-1},$$

Evaluating these matrix expressions gives

(12) $$\hat{\beta }=\frac{\sum w_{i}^{*}Y_{i}}{\sum w_{i}^{*}},\;$$

and

(13) $$\mathrm{Var}(\hat{\beta })=\frac{1}{\sum w_{i}^{*}},\;$$

so that Equations   (12) and (13) are identical to Equations   (2) and (3) ; the only cosmetic difference is that $\hat{\mu }$ is now denoted by the intercept $\hat{\beta }$. Furthermore, the normal distribution is used for making inferences under both this WLS regression model and the conventional method for meta‐analysis. Hence, making inferences under the random‐effects model for meta‐analysis using the conventional method is equivalent to making inferences under this WLS regression model.

### 4.2 WLS regression and the Hartung‐Knapp method for meta‐analysis

Now let us consider a second WLS regression model where $\epsilon_{i}∼N\left(0,k\left(s_{i}^{2}+{\hat{\tau }}^{2}\right)\right)$, so that the error variances are now assumed to be known up to a constant of proportionality, and we otherwise make the same assumptions as in the previous regression model. We obtain the same estimate $\hat{\beta }$ in the previous WLS regression model from Equation   (12); this is because we have already established that the same estimates of the regression parameters are obtained from the two types of WLS regression models. However Equation   (10), with the substitution of the estimate of *k* from equation  (11), now gives

(14) $$Var(\hat{\beta })=\frac{1}{n-1}{\hat{\epsilon }}^{T}W\hat{\epsilon }{\left(1^{T}W1\right)}^{-1}=\frac{\sum w_{i}^{*}{\left(Y_{i}-\hat{\beta }\right)}^{2}}{n-1}\frac{1}{\sum w_{i}^{*}},\;$$

where we have taken *p*=1 because there is one regression parameter (the intercept). Furthermore, both the WLS regression model with error variances known up to a proportionality constant and the Hartung‐Knapp method for meta‐analysis use the *t* distribution with *n*−1 degrees of freedom when making inferences for *β* (because *p*=1) and *μ*, respectively. The expression for $\mathrm{Var}(\hat{\beta })$ in Equation   (14) is, to within the same type of cosmetic differences as described above, the same as Var( $\hat{\mu }$) in Equation   (5) for the Hartung‐Knapp method for meta‐analysis. Hence, the Hartung‐Knapp method under the random‐effects model for meta‐analysis is equivalent to the WLS regression model where the error variances are known up to a constant of proportionality. The connections between these two models is further clarified by the observation that the quadratic form ${\hat{\epsilon }}^{T}W\hat{\epsilon }/(n-1)$ in Equation   (14) is equal to *H* ^*2^ in Equation   (5); from Equation   (11), we have $\hat{k}=H^{*2}$.

### 4.3 Random‐effects meta‐regression

All of the methods for WLS regression in Sections  3, and in particular Equations   (7), (8), (9), (10), and (11), apply in the WLS regression model for a more general regression where ***X*** is not given by **1** (the case where ***X***=**1** was examined to derive the results for meta‐analysis in the absence of covariates). We now apply the theory of WLS regression to random‐effects meta‐regression.^1^, ^2^, ^40^, ^41^ The random‐effects meta‐regression model is a generalisation of model (1)

(15) $$Y_{i}=\beta_{0}+\sum_{j=1}^{q}\beta_{j}x_{ij}+\varsigma_{i}+\delta_{i},\;$$

where *x*_*ij*_ is the value of the *j*th study level covariate in the *i*th study; *β*_0_ is the model intercept and the parameters *β*_*j*_, *j*=1,…,*q*, are the regression coefficients associated with the *q* study level covariates. We continue to assume *ς*_*i*_∼*N*(0,*τ* ^2^) and $\delta_{i}∼N(0,s_{i}^{2})$, but *τ* ^2^ is now referred to as the residual between‐study variance.

We will show, using the theory in Section  3, that random‐effects meta‐regression models can be fitted as WLS regressions where we do not simplify matters by taking ***X***=**1**. This extends the equivalences that we have established to the random‐effects meta‐regression setting. The matrix ***X*** therefore now also contains information on the covariates in additional columns. The only other distinction between the random‐effects meta‐regression and meta‐analysis model is that we continue to define ***W***=diag $\left(1/\left(s_{i}^{2}+{\hat{\tau }}^{2}\right)\right)$ but *τ* ^2^ but is now estimated under the meta‐regression, rather than under the meta‐analysis, model.

#### 4.3.1 WLS regression and the conventional method for random‐effects meta‐regression

The conventional method for meta‐regression is simply a WLS regression model where the weights are given by $1/(s_{i}^{2}+{\hat{\tau }}^{2})$, which are treated as known to be the reciprocals of the total study variances.^20^ Knapp and Hartung^20^ discuss the use of a variety of alternative statistical distributions when making inferences, but the first possibility they mention is the standard normal distribution. Upon deciding to use the standard normal distribution, the conventional method for meta‐regression is therefore equivalent to a known variance and independent errors WLS regression (Section  3.1) with ***W***=diag $\left(1/\left(s_{i}^{2}+{\hat{\tau }}^{2}\right)\right)$.

#### 4.3.2 WLS regression and the Hartung‐Knapp method for random‐effects meta‐regression

From Equations   (10) and (11), $\mathrm{Var}(\hat{𝛃})$ from the WLS regression described in Section  4.3.1 is multiplied by $\hat{k}=(1/(n-p)){\hat{\epsilon }}^{T}W\hat{\epsilon }=\sum w_{i}^{*}{\left(Y_{i}-{Ŷ}_{i}\right)}^{2}/(n-p)=H^{*2}$ to obtain the corresponding $\mathrm{Var}(\hat{𝛃})$ under the model where the error variances are known up to a constant of proportionality. This *H* ^*2^ is a generalisation of *H* ^*2^ in Equation   (5) for random‐effects meta‐regression where the average effect size $\hat{\mu }$ is replaced by the fitted values of the model ( ${Ŷ}_{i}$).

For this second WLS regression model to be equivalent to the Hartung‐Knapp method for random‐effects meta‐regression, the Hartung‐Knapp method must therefore also multiply conventional variances of $\hat{𝛃}$ by this more general *H* ^*2^. It must also use the *t*_*n*−*p*_‐distribution when making inferences, where *q*=*p*−1. This is exactly what the Hartung‐Knapp method does, and its details are fully explained (but using different notation to us) in Section  3 of Knapp and Hartung^20^ for a single covariate and in Viechtbauer et al^42^ in the more general situation when there are multiple covariates. Hence, also for the meta‐regression model holds that the conventional and Hartung‐Knapp methods are equivalent to WLS regression models where variances are known and known up to a constant of proportionality with ***W***=diag $\left(1/\left(s_{i}^{2}+{\hat{\tau }}^{2}\right)\right)$.

### 4.4 Summary

To summarise this discussion, the Hartung‐Knapp method for random‐effects meta‐analysis and meta‐regression can be implemented by fitting WLS regression models where the outcome data are the *Y*_*i*_, and the weights are the reciprocals of the estimated total study variances. This is because standard WLS regression software assumes that the variances are inversely proportional to the weights. The weights $1/(s_{i}^{2}+{\hat{\tau }}^{2})$ must however be manually specified. Hence,  *τ* ^2^ has to be estimated first by means of a random‐effects meta‐analysis or meta‐regression, so that the weights $w_{i}^{*}=1/(s_{i}^{2}+{\hat{\tau }}^{2})$ can be calculated. The conventional method for random‐effects meta‐analysis and meta‐regression can also be implemented by fitting closely related WLS regression models where the variances are treated as known.

## 5 EXAMPLES

We now numerically demonstrate the equivalence of the results from the conventional and Hartung‐Knapp methods for random‐effects meta‐analysis and meta‐regression and the two types of WLS regression models.

### 5.1 Computation in R

The conventional and Hartung‐Knapp methods, and the two WLS regression models, can be applied using R^39^ with the metafor^43^ and preloaded stats packages. Annotated R code illustrating how these models were fitted to both examples that follow is available via https://osf.io/y35m2/. Briefly, the random‐effects meta‐analysis and meta‐regression models were easily fitted to outcome data using the *rma.uni* function using the default restricted maximum likelihood (REML) estimator for estimating *τ* ^2^. This estimate of *τ* ^2^ was then incorporated in the weights that were computed with $1/(s_{i}^{2}+{\hat{\tau }}^{2})$. Hence, WLS regression models were subsequently fitted using the *lm* function and specifying the weights. Results from the second type of WLS regression model (Sections  3.2 and 4.3.2) were immediately obtained from R. By dividing the reported standard errors of estimated regression parameters from our second type of WLS regression model by $\sqrt{\hat{k}}$ as described by Thompson and Sharp,^40^ we obtained the corresponding standard errors from our first type of WLS regression model (Sections  3.1 and 4.3.1) where all variances are treated as fixed and known.

### 5.2 Example 1: Random‐effects meta‐analysis

We begin by applying all methods to a meta‐analysis concerning the effectiveness of open versus traditional education on student creativity where no covariates are included. This meta‐analysis contains 10 primary studies with standardized mean difference (Cohen's *d*) as effect size measure, and the data were obtained from Table 9 in Hedges and Olkin.^44^ Student's creativity in each primary study was measured by evaluating their ideas, figures, or drawings in response to a verbal or figural stimulus, and for each primary study was coded whether students were attending open versus traditional education. A positive standardized mean difference indicates that students' average creativity was larger in the open compared to the traditional education.

Analysis was performed using Hedges' standardized mean difference *g* as outcome data (the *Y*_*i*_ in model 1) in order to remove the small sample bias in Cohen's *d* (see Chapter 4 of Borenstein et al^2^). More specifically, Equations (4.23) and (4.24) of Borenstein et al^2^ were used to convert Cohen's *d* to Hedges' *g* and their within‐study variances. However, the exact (see Equation (6e) in Hedges^45^), rather than the approximate correction factor given in Equation (4.22) of Borenstein et al,^2^ was used in these two equations.

The first row of Table 1 shows the results of conventional meta‐analysis (Section  2.1) and the WLS regression with error variances assumed to be known (Section  3.1). Here, we show the estimated average effect size (Estimate), their standard errors (SE), the test statistics used to test the null hypothesis of no effect, the corresponding two‐sided *P *values, 95% CIs for the average effect size, the estimated between‐study variance obtained with the REML estimator,^1^ and the estimate of the proportionality constant *k*. The second row of Table 1 contains the same information for the Hartung‐Knapp method (Section  2.2) and WLS regression with error variances known up to proportionality constant *k* (Section  3.2). Table 1 shows that the conventional method for meta‐analysis produces the same results as our first WLS regression model and the Hartung‐Knapp method produces the same results as our second WLS regression model.

**Table 1:** Results of applying the random‐effects (RE) model using the conventional and Hartung‐Knapp (Modified) methods and weighted least squares (WLS) regression with error variances known (Known) and known up to a proportionality constant (Prop. constant) to the meta‐analysis on the effectiveness of open versus traditional education on student creativity

	Estimate	SE	Test Statistic	*P* Value	95% CI	${\hat{\tau }}^{2}$	$\hat{k}$
Conventional (RE)/Known (WLS)	0.246	0.176	*z* = 1.399	0.162	(‐0.099;0.591)	0.223	1
Modified (RE)/Prop. constant (WLS)	0.246	0.167	*t* = 1.477	0.174	(‐0.131;0.623)	0.223	0.896

*Note.* Estimate refers to the average effect size estimates, SE refers to the standard error, *p*‐value is the two‐sided *P* value, 95% CI refers to the 95% confidence interval, ${\hat{\tau }}^{2}$ is the restricted maximum likelihood estimate of the between‐study variance, and $\hat{k}$ is assumed to be one (denoted by $\hat{k}$ = 1) when using the conventional method/WLS regression with known error variances, and estimated using Equation  (11) when using the modified method/WLS regression where error variances are known up to a proportionality constant.

### 5.3 Example 2: Random‐effects meta‐regression

We also show the equivalence of the results from the methods using a random‐effects meta‐regression model with two covariates on the efficacy of the pneumococcal polysaccharide vaccine against pneumonia.^46^ Each participant was clinically and radiographically examined to determine whether a patient had pneumonia. The meta‐analytic dataset contains sixteen 2 x 2 frequency tables of randomized clinical trials on the efficacy of the vaccine. Moreover, two covariates were included in the model because healthy adults in low‐income countries and adults with a chronic disease in high‐income countries were predicted to be at greater risk of pneumonia than healthy adults in high‐income countries. Hence, these two covariates could affect studies' treatment effects. A negative log odds ratio implies that the vaccine was efficacious.

Log odds ratios (the *Y*_*i*_ in model 15) and their within‐study sampling variances were estimated using Equations (5.8), (5.9), and (5.10) as described in Borenstein et al.^2^ Two study level covariates were included in the random‐effects meta‐regression model. That is, two dummy variables were created that provide the *x*_*i*1_ and *x*_*i*2_ in model (15), so that *q*=2. The first of these covariates took the values 0 and 1 for randomized clinical trials that recruit participants with or without chronic illness, respectively. The second of these covariates took the values 0 and 1 for randomized clinical trials conducted in high or low‐income countries, respectively. Hence, the parameter *β*_0_ in model (15) is the average log odds ratio in randomized clinical trials conducted in high‐income countries that recruited patients with a chronic illness (reference category). The parameters *β*_1_ and *β*_2_ are log odds ratios that describe the differences in average log odds ratio of the reference category versus patients of randomized clinical trials conducted in high‐income countries that did not have a chronic illness and patients of randomized clinical trials conducted in low‐income countries with a chronic illness, respectively.

Table 2 shows that the same equivalences hold in the context of random‐effects meta‐regression. The results of conventional meta‐analysis and the WLS regression model with known error variances (first column) and Hartung‐Knapp method and the WLS regression model with error variances known up to proportionality constant *k* (second column) are numerically identical.

**Table 2:** Results of applying the random‐effects (RE) model using the conventional and Hartung‐Knapp (Modified) methods and weighted least squares (WLS) regression with error variances known (Known) and known up to a proportionality constant (Prop. constant) to the meta‐analysis on the efficacy of the pneumococcal polysaccharide vaccine against pneumonia

		Conventional (RE)/Known (WLS)	Modified (RE)/Prop. constant (WLS)
Estimate (SE)	$\hat{\beta_{0}}$	‐0.312 (0.178)	‐0.312 (0.179)
	$\hat{\beta_{1}}$	0.201 (0.281)	0.201 (0.284)
	$\hat{\beta_{2}}$	‐0.242 (0.286)	‐0.242 (0.289)
Test statistic (*P* value)	$\hat{\beta_{0}}$	*z* = ‐1.759 (0.079)	*t* = ‐1.742 (0.105)
	$\hat{\beta_{1}}$	*z* = 0.714 (0.475)	*t* = 0.707 (0.492)
	$\hat{\beta_{2}}$	*z* = ‐0.846 (0.397)	*t* = ‐0.838 (0.417)
95% CI	$\hat{\beta_{0}}$	(‐0.661;0.036)	(‐0.700;0.075)
	$\hat{\beta_{1}}$	(‐0.350;0.751)	(‐0.412;0.813)
	$\hat{\beta_{2}}$	(‐0.803;0.319)	(‐0.866;0.382)
${\hat{\tau }}^{2}$ or $\hat{k}$		${\hat{\tau }}^{2}$ = 0.146	${\hat{\tau }}^{2}$ = 0.146
		$\hat{k}$ = 1	$\hat{k}$ = 1.009

*Note.* $\hat{\beta_{0}}$ is the estimated model intercept; $\hat{\beta_{1}}$ and ${\hat{\beta }}_{2}$ are estimated log odds ratios that describe how the two study level covariates affect the average log odds ratio; SE refers to the standard error of $\hat{\beta_{0}}$, $\hat{\beta_{1}}$, and $\hat{\beta_{2}}$; *P* value is the two‐sided *P* value; 95% CI refers to 95% confidence interval; ${\hat{\tau }}^{2}$ is the restricted maximum likelihood estimate of the residual between‐study variance; and $\hat{k}$ is assumed to be one (denoted by $\hat{k}$ = 1) when using the conventional method/WLS regression with known error variances, and estimated using equation  (11) when using the modified method/WLS regression where error variances are known up to a proportionality constant.

Now that we have established the equivalences between the two types of methods for random‐effects meta‐analysis and meta‐regression, and the two WLS regression models, we will discuss the implications of these findings.

## 6 NEW INSIGHTS FROM THE NEW JUSTIFICATION FOR THE HARTUNG‐KNAPP METHOD

We have now established important links between two methods for meta‐analysis and two WLS regression models. Our main reason for establishing these connections is to provide further insight into the nature of the Hartung‐Knapp method. Two main types of additional insights are provided by our findings.

### 6.1 Intuition for why the Hartung‐Knapp method has been found to be more accurate in simulation studies

As we have already explained, the usual justifications of the conventional and Hartung‐Knapp methods explicitly require that all variances are treated as if fixed and known. We have also explained that simulation studies indicate that the Hartung‐Knapp method is more accurate, but both methods can be justified by the same random‐effects meta‐analysis model. Hence, except for the suspicion that the uncertainty in ${\hat{\tau }}^{2}$ may be taken into account by the Hartung‐Knapp method because a *t*‐distribution is used, there has previously been no intuitive reason for the better performance of the Hartung‐Knapp method. Our links with WLS regression models enable us to provide this intuition.

This is because we have established that inferences for the average effect from the Hartung‐Knapp method are equivalent to fitting an intercept only WLS regression model, with weights $w_{i}^{*}$, where the error variances are known *only up to a constant of proportionality*. We have therefore established a new type of justification for using the Hartung‐Knapp method for meta‐analysis. In this new justification, the variances are *not* assumed known. Although the strong assumption that the total study variances $s_{i}^{2}+{\hat{\tau }}^{2}$ are known to within a constant of proportionality is required ; this is a weaker assumption than the usual assumption that these are completely known. Our new justification for the Hartung‐Knapp method therefore helps to explain why it has been found to perform better in simulation studies. For example, in situations where the total variances $s_{i}^{2}+{\hat{\tau }}^{2}$ are likely to be positively biased, the Hartung‐Knapp method may be able to perform better if $E(\hat{k})<1$. That is, the likely positively biased total variance is in these cases scaled down if $\hat{k}<1$ (see Equation  10). The Hartung‐Knapp method may also be able to better describe real datasets where, for example, the estimated between‐study variance is much larger or smaller than the true value by compensating with a small or large $\hat{k}$, respectively. To summarise, the Hartung‐Knapp method has some potential to reduce the problems associated with the estimation of the variance components in meta‐analysis, albeit in a very direct and crude manner. The conventional method is not able to do this and so can be expected to perform worse, exactly as simulation studies have found.

### 6.2 Ad hoc adjustments to the Hartung‐Knapp method

We have already discussed the undesirable feature of the Hartung‐Knapp method that it may result in shorter CIs for the average treatment effect than the conventional method.^16^, ^19^ One solution to this has been the ad hoc suggestion to constrain *H* ^*2^ ≥ 1 in Equation   (5), so that $V_{\hat{\mu }}^{HK}\geq V_{\hat{\mu }}$. The use of quantiles from the *t* distribution by the Hartung‐Knapp method then ensures that the CI of this method is wider than the CI of the conventional method. However, it is hard to justify constraints such as this on any grounds other than a desire to be conservative or cautious when using the established justification of the Hartung‐Knapp method.

Our new justification of the Hartung‐Knapp method also provides insight concerning this issue and gives additional credence to the idea of placing constraints on *H* ^*2^. This is because usually when fitting WLS regression models using standard methods, we assume that the error variances are known to within a constant of proportionality *where we have no further information about the magnitude of the residual variance*. Hence, we may quite reasonably estimate *k* to be any positive number. However, in meta‐analysis we will usually have estimated both $s_{i}^{2}$ and *τ* ^2^ to at least a reasonable degree of precision, and so we know that $s_{i}^{2}+{\hat{\tau }}^{2}$ is approximately the variance of *Y*_*i*_. This implies that *k*≈1, but usually, when fitting WLS regression models we do not have this insight.

Furthermore, and as explained above, we have $\hat{k}=H^{*2}$. This suggests that we should consider constraining *H* ^*2^ to be close to one in the estimation. This gives, in particular, credence to the idea of constraining *H* ^*2^ ≥ 1 to prevent otherwise very small *H* ^*2^ resulting in artificially short CIs, but constraining *H* ^*2^ ≥ 1 is overly conservative^17^, ^20^ and our analysis suggests that we should consider constraining *H* ^*2^≈1, rather than *H* ^*2^ ≥ 1. Converting this suggestion to a recommendation for constraining *H* ^*2^ in a particular way is very difficult, because it depends on characteristics of the meta‐analysis (eg, the number of effect sizes included in a meta‐analysis).

Despite this, we can make one concrete recommendation. Jackson et al^19^ propose an approach that selects the CI based on the conventional or Hartung‐Knapp method depending on which is the widest (this is their second hybrid method). This conservative approach can also be implemented by constraining *H* ^*2^, because the widths of the CI of the conventional and Hartung‐Knapp method are equal to $2z_{\alpha /2}\sqrt{1/\sum w_{i}^{*}}$ and $2H^{*}t_{n-1,\alpha /2}\sqrt{1/\sum w_{i}^{*}}$, respectively. Hence, the CI from the Hartung‐Knapp method is the same as the conventional one if *H* ^*^=*z*_*α*/2_/*t*_*n*−1,*α*/2_, is shorter if *H* ^*^<*z*_*α*/2_/*t*_*n*−1,*α*/2_, and is wider if *H* ^*^>*z*_*α*/2_/*t*_*n*−1,*α*/2_. Taking the widest CI of the two methods is therefore equivalent to constraining *H* ^*^ ≥ *z*_*α*/2_/*t*_*n*−1,*α*/2_ when using the Hartung‐Knapp method. This constraint can also be expected to result in a conservative analysis since the widest of the two CIs is presented. However, this adjustment is less conservative than the one proposed by Knapp and Hartung^20^ where *H* ^*^ (or equivalently *H* ^*2^) is constrained to be greater than or equal to one. Hence, we suggest that any meta‐analysts who may have adopted the convention of constraining the scaling factor to be greater than one should consider instead applying the constraint *H* ^*^ ≥ *z*_*α*/2_/*t*_*n*−1,*α*/2_. This will also prevent very small *H* ^*2^ resulting in artificially small standard errors.

## 7 CONCLUSIONS

The Hartung‐Knapp method has been recommended for general use because it provides more accurate inferences (ie, coverage probabilities closer to the nominal coverage rate) for the average effect than the conventional random‐effects meta‐analysis method.^15^, ^16^, ^17^ The contribution of our paper to the literature is threefold. First, we have shown that the conventional and Hartung‐Knapp methods for random‐effects meta‐analysis and meta‐regression are equivalent to WLS regression models where the error variances are assumed to be known, and assumed to be known up to a constant of proportionality, respectively. In particular, this provides a new, and more insightful, justification of the Hartung‐Knapp method. By using standard methods for WLS regression models to motivate some of the main methods for meta‐analysis, we hope that this work will show that these methods are essentially just (albeit slightly adapted) standard statistical methods. Second, we provide intuition using this equivalence for why coverage of the CI based on the Hartung‐Knapp method has been found to be closer to the nominal coverage rate than the conventional method in simulation studies.^15^, ^16^, ^17^ Finally, we explain why this equivalence gives greater credence on placing ad hoc constraints on the scaling factor *H* ^*2^, and we therefore suggest that methods using a variety of such constraints are worthy of further consideration.

We do not recommend as ad hoc constraint *H* ^*2^ ≥ 1, as has previously been proposed.^20^ In situations where such caution is required, we suggest instead imposing the constraint *H* ^*^ ≥ *z*_*α*/2_/*t*_*n*−1,*α*/2_ that is less conservative and is tantamount to presenting the most conservative of the conventional CI and the CI of the Hartung‐Knapp method. Currently, only a limited number of papers^17^, ^20^ study the properties of the CI of the Hartung‐Knapp method when constraining *H* ^*2^ ≥ 1 but these papers do not consider alternative, and less conservative, constraints. Hence, future research could explore how the coverage probabilities, and other properties such as interval length, of the CI are affected by applying a variety of constraints on *H* ^*2^. Moreover, Jackson and Riley^47^ generalised the Hartung‐Knapp method to multivariate meta‐analysis where also a scaling factor similar to *H* ^*2^ is involved, so an opportunity for future research is also to explore whether statistical properties of the CIs with the Hartung‐Knapp method are improved if constraints are placed on this scaling factor.

Our new justification for the Hartung‐Knapp method opens doors for applying meta‐analysis models with standard statistical software for linear models, because the error variances are usually assumed to be known up to a constant of proportionality in this software. Hence, the random‐effects meta‐analysis and meta‐regression models can be fitted using popular linear model software packages (ie, SPSS,^37^ SAS,^38^ and R^39^) as long as an estimate for the (residual) between‐study variance is available that can be used to compute the weights. We suggest that the Hartung‐Knapp method is, in many respects, more closely related to other statistical methodologies than the more conventional approach. For example, the Hartung‐Knapp method is similar to the methodology applied in particle physics (for a discussion see Baker and Jackson^48^ and Jackson and Baker^49^) and in economics where meta‐analyses are usually conducted with WLS regression models without including an estimate of the between‐study variance in the weights.^50^ By using methods that are implemented in standard software and widely used, the Hartung‐Knapp method enables meta‐analysts to more directly use standard WLS regression model results and algorithms, for example, those that relate to model diagnostics and checking. Moreover, using the Hartung‐Knapp method does not limit the applicability of model diagnostics^51^ and graphical methods (eg, forest plot^52^, ^53^) that have been developed in the context of meta‐analysis. This is because the Hartung‐Knapp method's estimated average effect is the same as that of the conventional method whereas differences in the variance of this point estimate only have a minor influence on model diagnostics and graphical representations.

Our new justification applies to all types of data were the random‐effects meta‐analysis or meta‐regression model is used. However, the approximations made by these models are not necessarily very accurate in datasets that contain a small number of studies with small sample sizes.^6^, ^7^ The assumption that the total variances are known up to a constant of proportionality, as required by our new justification of the Hartung‐Knapp method, is also generally implausible in such situations. However, for the Hartung‐Knapp method to provide an improvement over the more conventional approach, this assumption merely needs to be more plausible than assuming that these variances are completely known. The Hartung‐Knapp method is therefore conceptualised as providing an improvement because it requires less implausible, rather than plausible, assumptions about our knowledge of the variance components.

Our new justification also emphasises the normality assumptions made by standard methods for meta‐analysis as these are explicitly made when presenting WLS regression models. Models that avoid normal within‐study approximations, for example, generalised linear mixed models for binary outcome data,^54^, ^55^ should be considered more often in application because, for example, inaccurate normal within‐study approximations can result in bias.^10^, ^56^ These more sophisticated models will result in a different point estimate as well as CI and have the potential to overcome concerns about biases that might result from making strong normality assumptions when using the conventional random‐effects model. However, the most appropriate way to use generalised linear mixed models for random‐effects meta‐analysis remains an open question that we do not attempt to address in this paper.

To summarise, we have provided a new justification for the Hartung‐Knapp method. This new justification requires that the total study variances are known only up to a constant of proportionality. This helps to explain why the Hartung‐Knapp method has been found to be more accurate than the conventional method and gives more credence to placing constraints on *H* ^*2^ when computing the variance of the estimated average effect. We suggest that our new justification of the Hartung‐Knapp method should replace the established one because it provides valuable additional insights and makes greater connections between methods for meta‐analysis and statistical methods more generally. We hope that our new insights will help to inform the meta‐analysis community as it determines which, if any, of the many alternative methods for meta‐analysis might ultimately replace the current approach.

## CONFLICT OF INTEREST

The author reported no conflict of interest.
